# Transformative Namaste Care: An Intervention Study Enhancing Quality of Life for People Living With Advanced Dementia

**DOI:** 10.1177/14713012251389433

**Published:** 2025-10-14

**Authors:** Sara Karacsony, Sharon Andrews, Melissa Abela, Maryam Rouhi, Thi Thuy Ha Dinh

**Affiliations:** 1 School of Nursing, UTAS Health, 3925 University of Tasmania, Australia; 2 Wicking Dementia Research and Education Centre, UTAS Health, 3925 University of Tasmania, Australia; 3 UTAS Health, 3925 University of Tasmania, Australia; 4 School of Nursing and Midwifery, 2541 Monash University, Australia

**Keywords:** quality of life, Namaste care, mixed methods, triangulation, intervention

## Abstract

**Background:** Few interventions specifically address the psychosocial care needs of people living with advanced dementia nearing the end of life in residential aged care facilities (RACFs). This study evaluated the effects of enhanced sensory care —touch, taste, smell, and social interactions—using the Namaste Care™ program on quality of life over a 12-week intervention period. **Methods:** Quality of life and agitated behaviours were assessed using two validated instruments (QUALID and CMI), at weeks 2, 4 and 12. Changes in scores were analysed using linear mixed effect models. Observational data on activities and their effects on residents were documented by Namaste Carers in session logs and mapped to a coding matrix corresponding to QUALID categories. Data were triangulated to provide a comprehensive picture of the intervention’s impact on residents’ quality of life. **Results:** Seven out of thirty residents screened completed the 12-week intervention. QUALID and CMAI scores significantly improved at the endpoint compared to baseline measurements. Observational data revealed residents enjoying social interactions, appearing calm and comfortable with Namaste Care alleviating agitation. **Conclusion:** Improved scores on the QUALID and CMAI were achieved within the first two weeks of the intervention, underscoring how enhanced care activities can quickly and positively influence mood states and behaviours, thereby enhancing quality of life. This is particularly important for residents in the advanced stages of dementia including those in the end-of-life phase. Many eligible residents missed out on this beneficial program that has no known negative outcomes.

## Background

People in the advanced stages of dementia are not able to engage in typical leisure or memory care activities because of difficulties with executive function and communication (1). The absence of meaningful engagement activities for people with advanced/advancing dementia can contribute to disturbed behaviour, depression, apathy, boredom and social isolation (Cohen-Mansfield et al., 2010; Michelet et al., 2022). A specialised program to improve the quality of life for these residents and to support families in maintaining connections with their loved ones has been championed by experts in the field (Smaling et al., 2018; Volicer, 2019) aligning with recommendations that multiple care components are needed to address the complex palliative care needs of this population (Kochovska et al., 2020; Van Der Steen et al., 2014). The Namaste Care^TM^ program, is a structured, non-pharmacological, psychosocial and person-centred program underpinned by a palliative approach that has been widely supported as a program that can improve living and dying for people with advanced dementia in Residential Aged Care Facilities (RACFs) (Bunn et al., 2018; Latham et al., 2020). The program caters to people experiencing severe cognitive and functional decline who have needs across physical, social, emotional and spiritual domains well before the end-of-life stage (Browne et al., 2021). Addressing quality of life is a fundamental goal of palliative care through the relief of suffering and treatment of pain and other problems, physical, psychosocial and spiritual (World Health Organization (WHO), 2024). Quality of life is identified as the most frequently endorsed biopsychosocial target for wellbeing in people receiving palliative care (Pakenham & Martin, 2024). The current evidence on palliative care interventions for individuals with advanced dementia is limited and inconclusive, particularly in terms of the impact on quality of life (Walsh et al., 2021). Evidence suggests that palliative care is largely provided as terminal care (last few days of life) and there has been limited research into models of care that seeks to provide palliative care in more holistic and proactive ways in RACFs (Volicer, 2019). A broader understanding of palliative care as non-medical supportive care that can provide equitable care for all people irrespective of diagnosis is advocated within a public health approach to end-of-life care (Abel & Kellehear, 2016).

Several studies have evaluated and demonstrated the positive effects of a Namaste Care program on quality of life for people living with advanced dementia in RACFs worldwide, including in Iranian nursing facilities (Amrollah Majdabadi Kohne et al., 2021; Rezapour-Nasrabad et al., 2023), United Kingdom (UK) care homes (Froggatt et al., 2020; Latham et al., 2020), long-term care homes in Canada (Kaasalainen et al., 2020; Yous et al., 2023), nursing homes in the Netherlands (Smaling et al., 2019) and in long-term care homes in the USA, where the program originated (Manzar & Volicer, 2015). Studies on the effectiveness of Namaste Care programs have also been conducted in the community setting, showing enhanced wellbeing of persons with dementia (Yous et al., 2022) and increased engagement and social interaction (Dalkin et al., 2020). In the hospital setting, patients with dementia receiving Namaste Care or an adapted version of it, also showed improved mood and positive responses engaging in stimulating activities (Yous et al., 2024) and improvement in quality of life (Ali et al., 2020). Effectiveness of the program has been demonstrated across a range of domains that impact on the quality of life for people with advanced dementia. Specifically, the deliberate use of a ‘loving’ touch’ approach as one of the key pillars of Namaste Care has been found to confer emotional, physical and observable mental health benefits for care recipients such as increased communication, social engagement, and connection with caregivers (Nicholls et al., 2013; Simard & Volicer, 2010; Smaling et al., 2022). There is also evidence that the Namaste Care Program^TM^ can result in a reduction in changed behaviours as expressions of unmet needs (Simard & Volicer, 2010; Stacpoole et al., 2015), and a decrease in the use of antipsychotic medications (Fullarton & Volicer, 2013; Manzar & Volicer, 2015; Simard & Volicer, 2010).

Our project aimed to implement the Namaste Care Program^TM^ as a new model of care to address gaps in palliative care for residents in the advanced stages of their illness, and to evaluate the program’s effects on participants’ quality of life, including effects on agitated behaviours. This research is crucial for the broader implementation of the program as a new model of care for those people living with advanced dementia.

This paper reports the effects on quality of life and behaviours of residents enrolled in the Namaste Care program at the end stage of the dementia trajectory.

## Methods

### Study Design

This project employed a four-stage implementation design adhering to an ethics-endorsed protocol.

Stage one focused on educating and training of participating staff, family and volunteers at participating sites, as well as setting up of the Namaste Care space.

Stage two involved setting up a Namaste Community of Care (CoC) at each site. The CoCs comprised key staff from the site who expressed an interest in championing and planning the daily program plus family caregivers/care partners and volunteers. Each CoC worked with the researchers to co-design an implementation plan for the roll out of the Namaste Program^TM^ at their service.

Stage three involved screening and recruitment of eligible residents to the program.

Stage four involved the program roll out by members of the Namaste CoC, daily delivery by Namaste Carers, and an evaluation of the intervention over a six-month period.

This paper focuses on Stages three and four: screening and recruitment of eligible residents and evaluation of the program. Outcome measures of quality of life and agitated behaviours of residents were assessed with structured questionnaires. Participant observations were conducted to examine the changes in moods and behaviours of residents during 4 occasions at baseline, 2 weeks, 4 weeks and 12 weeks.

### Setting, Participants, Sample Size

The study was conducted in three Residential Aged Care Facilities (RACFs) operated by a large not-for-profit organisation in Tasmania. Residents who met the eligibility criteria and would benefit from the Namaste Care intervention were identified by CoC members, including facility managers who were senior clinical registered nurses.

Inclusion criteria:

• A diagnosis of dementia
• Inability to actively participate in dementia activity/memory programs as assessed by a senior clinical nurse at the service
• Functional Assessment Staging of Alzheimer’s Disease (FAST) 6 or 7 – a senior clinical nurse confirmed the resident’s score and eligibility.

The target sample was thirty residents, with ten from each of the three participating sites. Participants were enrolled gradually over a six-month period to allow for the consent process, for residents to join the program when possible, as well as room capacity and other resource implications e.g. staffing, that naturally limit the number of residents who can comfortably be accommodated in a Namaste Care session.

### Namaste Care^TM^ Program Intervention

The program was designed to be undertaken in a group setting, involving family carers, staff and volunteers for its delivery. In its original format, the daily program is recommended for 2 hour sessions in the morning and afternoon seven days a week (Simard, 2022). In this project the program was delivered in a two-hour afternoon session only, as a pragmatic approach to introducing the Namaste Care program into the daily workflow of staff while ensuring their workload is manageable.

The program offers multi-sensory activities stimulating all the senses – tactile, olfactory, gustatory, auditory and kinaesthetic/proprioception - aimed at enhancing the quality of life of individuals with advanced dementia (1). It has two principles: a calm environment and a ‘loving’ (slow, unhurried) touch approach and pain assessment is integral (Simard, 2022). Namaste Care specifically stimulates a person’s senses to promote comfort and pleasure (Karacsony & Abela, 2021). Namaste Care has been specially developed for residents in the advanced stage of dementia, who may become agitated when there is too much stimulation (Fullarton & Volicer, 2013; Manzar & Volicer, 2015; Nicholls et al., 2013; Simard & Volicer, 2010; Smaling et al., 2022; Stacpoole et al., 2015). Key activities of Namaste Care include gentle face washing and face moisturising, shaving the “old-fashioned way”, hair combing/styling, hand and foot massage, range of motion exercises, reminiscence activities, interaction with seasonal scents, and other activities tailored to individual recipient preferences when these are known (Simard, 2022).

Commensurate with the Namaste Care Program protocol, participants were required to be screened for pain before each session using the organisation’s usual pain assessment tool to ensure they were comfortable before attending the session (Simard, 2022).

Throughout the sessions, a variety of multisensory activities were provided by staff and family e.g:

• **Expressive touch:** gentle face washing, hair combing and hand massage to communicate and connect with the residents
• **Snacks and beverages:** offering residents favourite/tasty foods and drinks to experience different tastes, textures and sensations
• **Engagement items:** incorporating significant or personalised items for individual residents, such as magazines and photography books, bubbles, and music/songs.

### Measured Outcomes and Instruments

Quality of life was assessed using the Quality of Life in Late-Stage Dementia (QUALID) (Weiner et al., 2000). The QUALID instrument measures quality of life across 11 domains: smiles, appears sad, cries, facial expression, physical, statements or sounds, irritability or aggression, enjoys eating, enjoys touch, enjoys interaction, appears emotionally calm. Each item is measured from 1-5, and all responses are summed, with possible scores range from 11 to 55, where 11 (lowest score) represents the highest quality of life. Psychometric testing of this instrument was further established by Resnick and colleagues (2018) who agreed on the reliability and validity of the QUALID scale, recommending adding pain into the domain ‘appears physically uncomfortable due to pain’ to acknowledge pain as an important aspect of quality of life. Some additional items that represent activities or interactions with staff and their satisfaction were also suggested because these aspects also influence quality of life (Resnick et al., 2018).

Agitated behaviours were assessed as a secondary outcome using the Cohen-Mansfield Agitation Inventory (CMAI) (Cohen-mansfield et al., 1989). This instrument consists of 29 agitated behaviours, each rated on a 7-point frequency, where “1” indicates the behaviour never occurs, and “7” indicates the behaviour occurs several times an hour on average. The 29 agitated behaviours each represent a group of diversified behaviours: physical/aggressive, physical/non aggressive, verbal/aggressive, verbal/non aggressive. Aggregation to better understand certain behaviours is suggested as being more meaningful than calculating a total score (Cohen-Mansfield, 1991).

Both the QUALID and CMAI measures were found to be useful measurements as both instruments detected changes at 4 weeks in a feasibility study on Namaste Care and were recommended outcome measures in future trials of this intervention (Froggatt et al., 2020).

### Data Collection and Analysis

The QUALID (Weiner et al., 2000) and CMAI scales were administered in an interview format to the Namaste carer (or Clinical Care Coordinator) who was most familiar with the resident during the intervention period. These staff were asked to respond to the questionnaires based on observations of the resident over the past seven days. The QUALID and CMAI were assessed at baseline, and at 2-, 4- and 12-weeks post intervention. Pain assessment was required as part of usual care processes before residents joined the daily program and was not an outcome of the intervention.

Data were input in SPSS version 29. The QUALID and CMAI scores were calculated using the sum of all item scores. Descriptive statistics were used to describe the sample using counts and frequencies for the categorical variables, and means, standard deviations for the continuous variables when possible. Linear mixed effects models were performed to examine the differences in QUALID and CMAI scores across four data collection occasions over 12 weeks. Time was defined as a fixed factor, as the QUALID and CMAI were expected to change over time, while the participants, the RACF they were residing in, and number of Namaste Care sessions they received during 12 weeks, and the average time of one Namaste Care session offered to them (calculated by averaging four observations) were added in the models as random effects. All fixed effects were considered significant when *p* ≤ 0.05. Although the sample size was small in this study, linear mixed effects models have demonstrated utility for analysing longitudinal data of as small as five individuals and so was appropriate to be used in this instance (Wiley & Rapp, 2019).

### Resident Observations and Analysis

The Namaste carer responsible for running the daily program observed the activities residents received and their reactions to these activities, indicating whether the person enjoyed the activities or not, and recorded this information using unstructured free text in a daily summary log until the end of Week 12. There was also a section to note any issues that arose during the session, facilitating handover to future Namaste Carers (Kendall, 2019), for example to exclude face massage for a particular resident when there was a note that “she refused face massage”.

The observational data were collected and mapped to the QUALID categories (Supplemental files A and B) to provide a more holistic insight of the intervention’s impact on quality of life. This mapping supported the identification of patterns and trends in residents’ moods and behaviours during the sessions that might not be fully captured by structured instruments like the QUALID. We undertook this mapping because observational data collected in naturalistic settings reflects real-world behaviours and experiences of participants within the context of their actual lived experiences (Salmon, 2014; Williams, 2025). Mapping of the observational data to the QUALID domains also enabled triangulation of the data sets with the qualitative data enhancing the credibility of the quantitative results.

## Results

From the three sites recruited, the program was implemented at two sites as one withdrew due to staffing issues. The Namaste Care program was scheduled to run for 2 hours per day for 12 weeks. Beyond this time, residents could still continue their access to the Namaste Care program if they wanted to. Ten residents were initially recruited across the two sites. There was attrition of three residents: two residents died during the intervention period and one resident enrolled late in the study and did not continue in the program after baseline data collection. Thus, data was analysed for seven residents.

### Demographic Characteristics

All residents were female except for one, mostly in their late 80s and 90s. Five participants had a diagnosis of dementia and five others were documented as unspecified dementia/short-term memory loss in lieu of formal diagnosis. While three residents attained a FAST of 6 indicating severe impairments in function, they were able to engage in words. Most residents scored a FAST of 7, including the most advanced dementia with two residents scoring a FAST of 7e indicating loss of ability to smile.

A total of 7 residents received the intervention across all time points while three residents received some Namaste Care sessions but did not continue beyond baseline, week 1 and week 10 respectively. Two participants received additional sessions after week 12. The average duration of Namaste Care sessions among participants was calculated as approximately 61.03 minutes per session (Table 1).

**Table 1.** Demographic Data and Clinical Characteristics

ID	Age	Gender	Diagnosis	Stage of dementia (FAST)	No. of NC sessions	Adjusted average length of NC
01^a^	90	F	Unspecified dementia	6d or 6e	41	98.3
02^c^	89	F	Alzheimer’s disease	7a, 7b	31	88.75
03	84	F	Short term memory loss	7c or7d	28	82.5
04	89	F	Alzheimer’s disease	6d or 6e	27	80
05	92	F	Vascular dementia	6d or 6e	19	81.6
06^a^	82	F	Vascular dementia	7a or 7b	3	60
07^b^	92	F	Short term memory	7c or 7b	2	60
08^c^	71	M	Frontal temporal dementia	7e	41	41.25
09^a^	87	F	Unspecified dementia	7c or 7d	56	56.25
10^d^	78	F	Unspecified demetia	7e	56	16.25

^a^Residents died at week 10/week 1.

^b^Resident did not continue after baseline assessment.

^c^Residents continued to receive 5 and 18 NC sessions following week 12.

^d^Had bedside NC.

### Effectiveness of Namaste Care Intervention on Dementia Quality of Life

**Figure 1.**
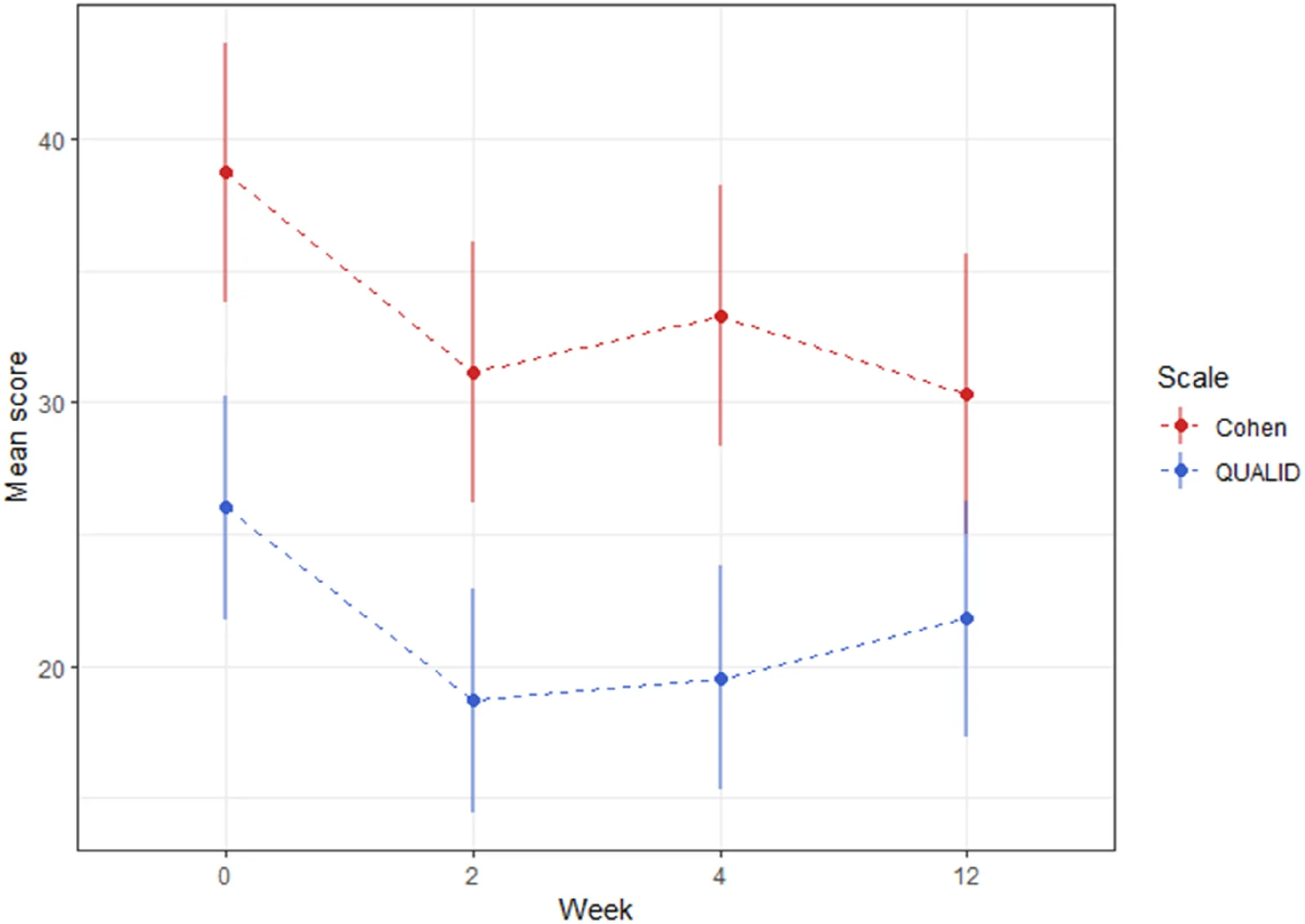
Estimated QUALID and CMAI Scores Were Estimated at Each Time Point Using Linear Mixed Effects Models. *Note.* Lower Scores Show Improvements in Quality of Life and Agitated Behaviours

The QUALID average scores at 4 occasions (T1, 2, 3, 4) were 25.2 (SE = 2.17), 17.9 (SE = 2.17), 18.8 (SE = 2.17) and 20.8 (SE = 2.27), respectively. The QUALID scores significantly reduced over 12 weeks. Although the endpoint QUALID was not significantly reduced compared to baseline, the significance level was close to 0.05 (*P* = 0.07, see Table 2 and visual display Figure 1). Missing data may have contributed to this result as, noticeably, there was one resident who did not have an endpoint QUALID score that could have impacted on this.

**Table 2.** Effectiveness of Namaste Care on Quality of Life (QUALID)

Model	Adjusted coefficient	95% CI	*P* value
Intercept	25.3	[20.4, 30.2]	<0.001
Time			
Week 2 vs baseline	−7.29	[−11.8, −2.70]	0.004
Week 4 vs baseline	−6.43	[−11.0, −1.85]	0.009
Week 12 vs baseline	−4.37	[−9.15, 0.40]	0.07

### Effectiveness of Namaste Care Intervention on Agitation Behaviours

The CMAI average scores at 4 occasions (T1, 2, 3, 4) were 38.7 (SE = 2.37), 31.1 (SE = 2.37), 33.3 (SE = 2.37) and 30.3 (SE = 2.55), respectively. The CMAI scores significantly reduced over 12 weeks (*P* = 0.01), with the highest reduction seen at end-point measurement (see Table 3 and visual display Figure 1).

**Table 3.** Effectiveness of Namaste Care on Agitated Behaviours (CMAI)

Model	Adjusted coefficient	95% CI	*P* value
Intercept	38.7	[33.8, 43.6]	<0.001
Time			
Week 2 vs baseline	−7.57	[−13.8, −1.27]	0.02
Week 4 vs baseline	−5.43	[−11.7, 0.87]	0.09
Week 12 vs baseline	−8.41	[−15.0, −1.8]	0.01

### Observation Data Findings

Observational data mapped to the QUALID categories shown in Tables 2 and 3 (Supplemental file) showed most interactions within the Namaste Care sessions mapped to ‘Enjoy interacting or being with others’ followed by ‘Appears calm and comfortable’. All residents were seen to interact and engage with activities or staff or were perceived as enjoying the ‘company’ of others. Staff described interactions in terms of; ‘loves’, ‘enjoyed’. For example,

“She loves the Namaste it’s the one opportunity she gets to have one on one. She loves female company, and the talk.”

Personalisation of activities was also evident in this category, such as, showing a London picture book to an English-born resident, which elicited positive responses and conversations about the person’s past:

“She was happy and talked about her journey to London”.

“ .. had wonderful conversations all about her past.”

Residents were mostly perceived as calm and relaxed during the program. Some were engaged in an activity e.g. rolling wool, or simply looking around whereas they had been previously sleepy as follows:

“In the beginning, he was sleepy but after a while he was awake”.

and,

“Happy, calm, eyes looking around, enjoyed bubbles”.

Some data was mapped to ‘appears emotionally calm and comfortable’, even if agitation was described early in the session because at the end of the session the resident’s emotional state was changed and they were settled:

“She is bit agitated but after having massage and drinks [is] looks happy and relaxed”.

However, there were a few instances of the resident appearing to remain agitated:

“Is ok doing all the care she likes hand & feet massage but sometimes she is bit agitated, maybe tired”.

Four segments of data mapped to ‘makes statements or sounds that suggest discontent, unhappiness, or discomfort’, but also to the settling effects that were achieved, such as:

“Always asking to put her in bed and take her to toilet. But talked to her and mentioned her about the session and she was alright & had a little chat together”.

and,

“Calm, reading magazine but keep saying ‘am I going to bed?’ in middle of session”.

However, no data mapped specifically to ‘appears physically uncomfortable.’ or ‘cries’ or ‘has a facial expression of discomfort’.

Very little data mapped to ‘enjoys eating’ although residents were offered nourishments and beverages at each session, including chocolate and ice cream.

Data was also evident for ‘smiles’ despite two residents classified as 7e against the FAST:

“The mood of the patient at the end of first session was a small smile”.

and,

“I spent about half an hour with her on my own today. She became very chatty with me and smiled a lot”.

Data related to ‘enjoys touching/being touched’ was recorded in a positive way, as follows:

“The first timeline, the resident never initiate touching, but the second timeline (WK2), he initiate touching”.

and,

“Resident observed to be relaxed, responsive to his slow, gentle hand and shoulder massage”.

Another resident was described as *“very tired today but enjoyed her massage.”*

The data reveals staff members attuned to the resident’s presentation, mood fluctuations and responsiveness to care activities.

## Discussion

Our results showed notable improvements in both QUALID and CMAI scores for all residents who received the 12-week intervention compared to baseline measurements. While there was variability in the number of Namaste Care sessions and overall duration of session time for residents (the majority only received about an hour of Namaste Care for each session), the intervention showed positive results for participating residents. Our findings show improved scores on the QUALID scale, specifically in terms of responsiveness, engagement and communication with staff, as reported in other studies (Amrollah Majdabadi Kohne et al., 2021; Froggatt et al., 2020; Latham et al., 2020). We also had evidence of comfort, enjoyment and relaxation and a reduction in agitated behaviours as measured by the Cohen-Mansfield Inventory of Agitation (Cohen-mansfield et al., 1989; Froggatt et al., 2020). Importantly, we observed these positive effects within two weeks of the program commencement unlike other studies that show QUALID scores were responsive to change over four and 24 weeks (Froggatt et al., 2020; Kaasalainen et al., 2020). While no further improvements on the QUALID were found over the intervention period of 12 weeks, possibly due to factors such as illness or events impacting the resident’s wellbeing on the day of data collection, the relatively short study timeframe yielded a sustained improvement on QUALID scores from baseline, such that we can assert the effectiveness of Namaste Care as a short-term intervention and one that is applicable at end of life. Contrary to existing studies, we did not exclude residents unable to join the group program instead offering Namaste Care at the bedside so showing its applicability for an end-of-life population who maybe too unwell or unable to join a group program (Froggatt et al., 2020).

This study makes a unique contribution to the literature by triangulating qualitative observational data to illustrate the intervention’s effects on residents’ mood, behaviours and activities they enjoyed. These observations support the improvements in QUALID and CMAI scores, showing that simple sensory activities, such as gentle face, hand and foot massages, sensory items such as wool, and other activities like observing bubbles, eating and/or drinking and staff interactions, often led to increased relaxation, engagement and displays of happiness. We argue that these markers are, in fact, reflecting quality of life, as seen in other studies (Latham et al., 2020; Smaling et al., 2022). Although existing evidence for improvements in agitation and quality of life from tailored activities for people with advanced dementia is modest (Möhler et al., 2023), this study’s observational data captured positive effects from such activities that clearly confirm the instrument data. Consistent with other studies (Kaasalainen et al., 2020) that investigated the effects of the Namaste care program, observations also revealed that residents’ mood and behaviours were such that residents looked ‘happy’, ‘laughed’ and even start to sing. Our mapping of observational data to the findings of validated instruments offers a novel and rigorous layer of interpretation, adding depth and credibility to our findings by capturing the lived experiences and emotional responses of participants. A key strength of our data collection lies in the continuous observation of residents throughout the program, during which their mood states, responses, and even periods of sleep were carefully noted. This sustained attention stands in contrast to the task-oriented routines often found in aged care settings, where individuals with advanced dementia may be left unmonitored for extended periods (F. Bunn et al., 2018). Our approach not only enhanced the quality of data but may also reflect an enhancement of care rarely afforded to this population (F. Bunn et al., 2018). In particular, staff provided Namaste Care activities tailored to individuals with advanced dementia which were not part of usual care. This personalisation was possible because of the dedicated time and space for the Namaste program which primarily enabled highly person-centred care outside of the busy workflow. Positive responses were especially noted to touch and social interaction contributing to improved mood and relaxation in the staff observational data, however such responses are not captured in the metrics of the QUALID. One resident, initially unresponsive to touch began initiating touch by week 2, perhaps indicating a growing comfort with physical and social interaction. While this data is only for one resident, it is highly important because it confirms existing theory on the program providing structured access to social and physical stimulation (F. Bunn et al., 2018).

Previous studies have focused on optimal ‘dose’ and ‘coverage’ of interventions for addressing palliative care needs in advanced dementia (Kochovska et al., 2020) and/or program fidelity and feasibility of adopting the ‘gold standard’ dose of two daily sessions per day (Froggatt et al., 2018; Kaasalainen et al., 2020). While longer duration and increased frequency of sessions are likely to yield greater benefits to residents, our findings show that even a condensed, once-daily program produced immediate positive and sustained benefits. Residents displayed noticeable improvements in mood and engagement during most sessions—except when sleepy—regardless of how many sessions they attended or their duration. These results highlight that meaningful impact is possible, even when practical constraints limit full adherence to the 'gold standard’ model (Salvi et al., 2025).

Further, measuring quality of life (QoL) in people with cognitive impairment is challenging, as they cannot provide subjective data and proxy input may be inaccurate, hence this is why we have used the observations documented following each session. Our findings show that most observational data mapped positively to categories like Enjoyment and Appearing calm and comfortable, while some categories such as Cries were not observed. This aligns with other studies (Resnick et al., 2007) where some categories were easier to endorse than others supporting the recommendation for easier items and/or additional items (and/or observational data) to clearly show the effects of activities or interactions with trusted staff on residents’ quality of life. By including observational data, it is more straightforward to differentiate a positive mood from one that is sad when visible effects are evident. We maintain that any wellbeing activity that raises a smile, elicits laughter or a song is an indicator of enjoyment and is a more accurate proxy of quality of life and it is irrelevant whether the activity is personalised or not (Mohler et al).

Unlike previous studies in which participants with severe dementia were more engaged in activities related to nourishments and beverages (Cohen-Mansfield et al., 2012; Kaasalainen et al., 2020; Möhler et al., 2023), we found participants were more engaged in one-on-one activities and, to some extent, being in the company of others. Wellbeing priorities during the end-of-life phase for people receiving palliative care include sustained and present-moment relationships and addressing quality of life, which is the most frequently endorsed biopsychosocial target of palliative care patients (Pakenham & Martin, 2024). For people with advanced dementia, quality of life is paramount as cognitive improvements or maintenance of function are no longer the goal. Quality interpersonal connections, a key component of quality of life, cannot be easily quantified, raising the question of how to measure psychosocial benefits in advanced dementia. The smiley face included in the unstructured observational data attest to benefits to staff of seeing residents enjoying program activities, also evident in other studies (Kaasalainen et al., 2020; Smaling et al., 2022) confirming earlier findings of Namaste Care providing opportunities for enhanced interpersonal engagement (Bunn et al., 2018).

Although many residents were eligible for participation, the majority were not recruited to the intervention. The reasons for this varied, including staffing issues at the facility, communication barriers, and challenges with the proxy consent process. While these factors are beyond the scope of this paper, they have potential implications for residents’ quality of life at end of life. Missed opportunities to focus care on enjoyment and comfort can lead to apathy, boredom and loneliness among residents in aged care facilities, including those at end of life (Cohen-Mansfield et al., 2010). These residents represent an underserved population when it comes to interventions with no negative impacts (Latham et al., 2020) beyond attention to pressure area care (Kaasalainen et al., 2020) and clear psychosocial benefits. The impact on individuals is significant, as engagement in (meaningful) activities can provide distraction and improve wellbeing, even in the short term.

### Strengths and Limitations of Study

This mixed methods study triangulated the two data sets to provide a more holistic overview of the effects of the Namaste Care program on residents’ quality of life. Triangulation of data enhanced the credibility of QUALID scores, supporting the objective QUALID data with real time observations from Namaste Carers providing the daily sessions. We also note potential observer bias due to the dual role of Namaste Carers as both providers of the intervention and the observational data. However, given that dementia symptoms and their impact on quality of life can vary widely, the observational data was valuable in showing the variability in a person’s mood, and variations in observers’ descriptions and interpretations reducing the risk of bias from one data source only.

The small sample is a study limitation as is the absence of a control group that would enable clearer definitive causal conclusions about the intervention’s effects. The small sample size and the specific organizational setting also limit generalizability. However, in conjunction with the extant literature, we are confident this sample does represent the larger population of people living with advanced dementia who would be eligible for this intervention. This work provides a solid foundation for larger studies that can clearly show the effectiveness of this intervention. With more participants, greater quantity of observational data could be linked to the QUALID categories thereby further substantiating the conceptual domains of quality of life in this group.

## Conclusion

The immediate and sustained benefits from the Namaste Care intervention suggest that the program can have a significant impact on the quality of life of individuals who are in the latter stages of their dementia illness, even in the short-term. Further, the focus on the individual’s engagement and enjoyment offers a practical and relatable measure of success of the program's implementation, as demonstrated by mapping of detailed observational data in this study and its alignment with QUALID domains. By focusing on a population that often experience significant gaps in holistic aspects of palliative care, this study underscores the effectiveness of simple sensory engagement and social interactions. It highlights the need for a specialised program to improve quality of life for people with advanced dementia right to the end of life. This group remains an underrepresented and underserved population in both research and interventions aimed at improving quality of life.

These aspects make the study a valuable contribution to the field, offering further insights and evidence to support the broader adoption of the Namaste Care Program across RACFs where possible.

## Supplemental Material


Supplemental Material - Transformative Namaste Care: An Intervention Study Enhancing Quality of Life for People Living With Advanced Dementia
Supplemental Material for Transformative Namaste Care: An Intervention Study Enhancing Quality of Life for People Living With Advanced Dementia by Sara Karacsony, Sharon Andrews, Melissa Abela, Maryam Rouhi, Thi Thuy Ha Dinh in Dementia.

## Data Availability

Data is available on reasonable request to the principal author.*
