# Supplementary material for: Transformative Namaste Care: An Intervention Study Enhancing Quality of Life for People Living With Advanced Dementia
Source: Dementia (London). 2025 Oct 14;25(7):1628–44. doi: 10.1177/14713012251389433 (PMC13569905; doi:10.1177/14713012251389433)
Supplement: Supplementary file 1 [file sj-pdf-1-dem-10.1177_14713012251389433.pdf]

Supplementary_file_A

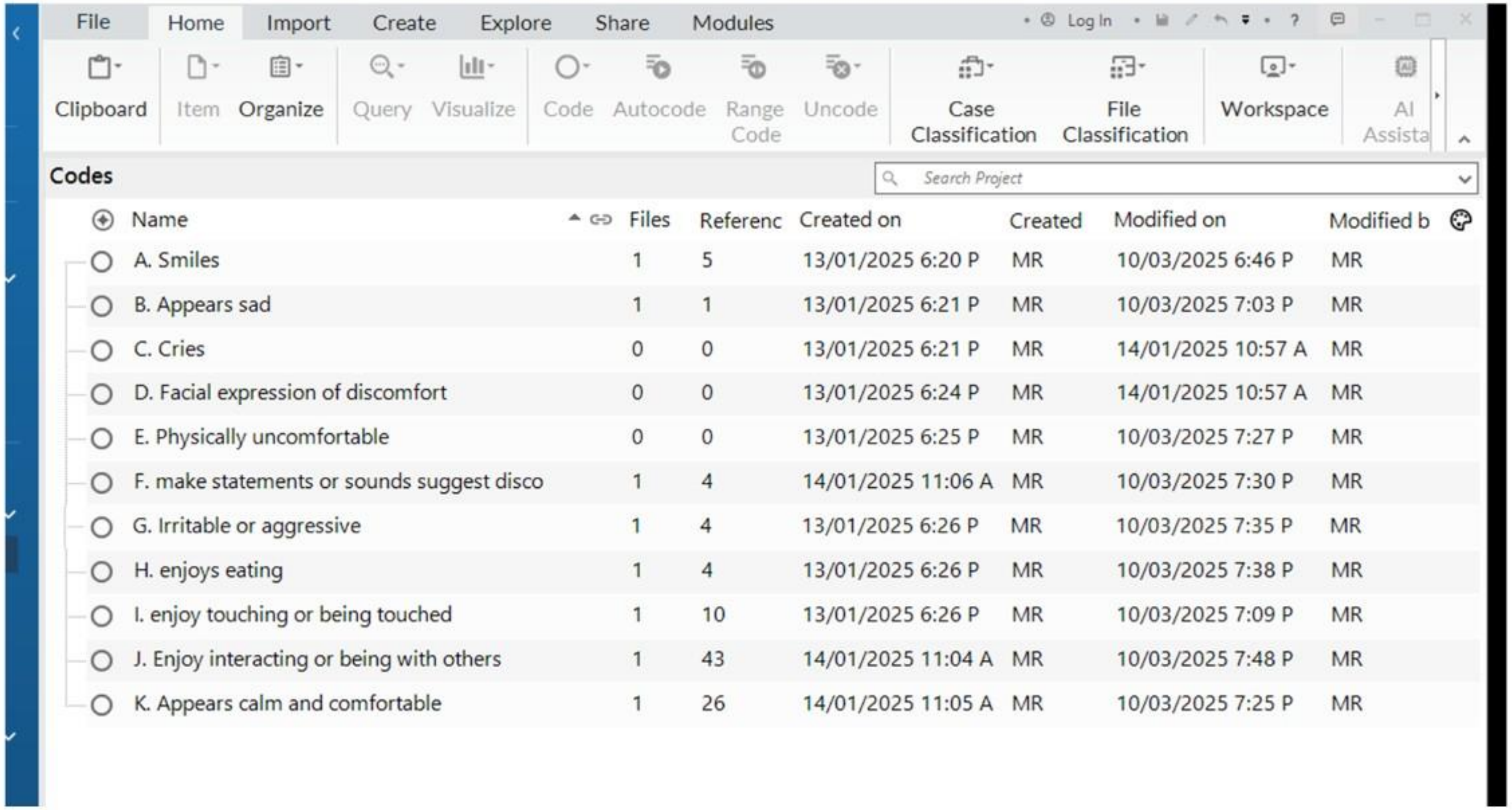

Name	Files	Referenc	Created on	Created	Modified on	Modified b
A. Smiles	1	5	13/01/2025 6:20 P	MR	10/03/2025 6:46 P	MR
B. Appears sad	1	1	13/01/2025 6:21 P	MR	10/03/2025 7:03 P	MR
C. Cries	0	0	13/01/2025 6:21 P	MR	14/01/2025 10:57 A	MR
D. Facial expression of discomfort	0	0	13/01/2025 6:24 P	MR	14/01/2025 10:57 A	MR
E. Physically uncomfortable	0	0	13/01/2025 6:25 P	MR	10/03/2025 7:27 P	MR
F. make statements or sounds suggest disco	1	4	14/01/2025 11:06 A	MR	10/03/2025 7:30 P	MR
G. Irritable or aggressive	1	4	13/01/2025 6:26 P	MR	10/03/2025 7:35 P	MR
H. enjoys eating	1	4	13/01/2025 6:26 P	MR	10/03/2025 7:38 P	MR
I. enjoy touching or being touched	1	10	13/01/2025 6:26 P	MR	10/03/2025 7:09 P	MR
J. Enjoy interacting or being with others	1	43	14/01/2025 11:04 A	MR	10/03/2025 7:48 P	MR
K. Appears calm and comfortable	1	26	14/01/2025 11:05 A	MR	10/03/2025 7:25 P	MR

Supplementary_file_B. Mapping of unstructured observational data to the Quality of Life in Late-Stage Dementia (QUALID) Scale

Smiles<br>1. spontaneously once or more each day<br>____<br>____	*1. The mood of the patient at the end of first session was a small smile. RV01*	***Appears physically uncomfortable - he/she squirms, writhes, frequently changes position***	

2. spontaneously less than once a day<br>____ 3. only in response to external stimuli; at less once a day<br>____ 4. only in response to external stimuli; less than once a day<br>rarely, if at all	2. *Looks so happy doing the care she is singing, laughing and talking very well. FR01*<br>3. *Looks so happy she told me about her family & she singing, laughing very well talking about her past. FR01*<br>4. *Resident is very happy doing the care. FR01*<br>5. *I spent about half an hour with her on my own today. She became very chatty with me and smiled a lot. FR02*<br><br>*Instances of residents smiling or 'looking happy' which may be interpreted as a smile*	____ 1. rarely or never<br>____ 2. less than once a day<br>____ 3. at least once a day<br>____ 4. nearly half theay<br>most of the day	
Appears sad<br>____ 1. rarely or never<br>____ 2. only in response to external stimuli; less than once a day<br>____ 3. only in response to external stimuli; at least once a day<br>____ 4. for no apparent reason less than once each day<br>for no apparent reason once or more each day	1. *She was a bit sad starting but after the care when looks a bit happy. FR01*	***Makes statements or sounds that suggest discontent, unhappiness, or discomfort*** *(complains, groans, screams)*<br>1. rarely or never<br>____ 2. only in response to external<br>____ stimuli; less than once a day<br>____ 3. only in response to external stimuli; at least once a day<br>____ 4. without cause less than once a day<br>without cause once a day	1. *Always asking to put her in bed and take her to toilet. But talked to her and mentioned her about the session and she was alright & had a little chat together.RV02*<br>2. *Calm, reading magazine but keep saying 'am I going to bed?' in middle of session. RV02*<br>3. *Wasn't very well within herself today. didn't have lunch, wasn't feeling it at all. FR01*<br>4. *She allowed to do hand &feet massage but she refused face massage. She likes to drink she said she doesn't like bit loud music FR07*
Cries		***Is irritable or aggressive***	1. *She is bit agitated but after*

____ 1. rarely or never<br>____ 2. only in response to external stimuli; less than once a day<br>____ 3. only in response to external stimuli; at least once a day<br>____ 4. for no apparent reason less than once each day<br>____ for no apparent reason once or more each day		*(becomes angry, curses, pushes, or attempts to hurt others)*<br>____ 1. rarely or never<br>____ 2. only in response to external stimuli; less than once each day<br>____ 3. only in response to external stimuli; at least once each day<br>____ 4. without cause less than each day<br>____ without cause once or more each day	*having massage and drinks [is] looks happy and relaxed. FR02*<br>*2. Bit agitated doing the care. When daughter try to massage her, she not happy try to grab her hand. FR02*<br>*3. Is ok doing all the care she likes hand & feet massage but sometimes she is bit agitated, maybe tired. FR02*<br>*4. Looks bit agitated sometime. She refused to have drinks & snack but she allow massage her hand, feet & face .. After that, she is relaxed FR02*<br>*5. She would like to wander around the room*<br>*She is ok for 30 min after that she want to wander.FR07*
***Has a facial expression of discomfort**- appears unhappy or in pain (looks worried, grimaces, furrowed or turned down brow)*<br>____ 1. rarely or never<br>____ 2. less than once a day<br>____ 3. at least once a day<br>____ 4. nearly half the day<br>____ most of the day		***Enjoys eating***<br>____ 1. at most meals and snacks<br>____ 2. twice a day<br>____ 3. at least once a day<br>____ 4. less than once a day<br>____ rarely or never	*1. Active, enjoyed ice-cream and reading magazine. RV02,*<br>*2. Had a wonderful time, enjoyed chocolate very much and we talked about Christmas FR06 [resident died during intervention]*
Enjoys touching/being touched<br>____ 1. almost always; almost always initiates touching<br>____ 2. more than half the time; sometimes initiates touching<br>____ 3. half the time; never initiates	*1. The first timeline, the resident never initiate touching, but the second timeline (WK2), he initiate touching.*<br>*2. Resident observed to be relaxed, responsive to*	***Enjoys interacting or being with others***<br>____ 1. almost always: almost always initiates interaction with others<br>____ 2. more than half the time; sometimes initiates	*1. Resident reacted to the song, comfort care and bubble item used. RV01*<br>*2. Sometimes open eyes looking around; sometimes sleeping RV01*<br>*3. Slept for first half session but*

touching, but does not resist touching<br>____ 4. less than half the time; often or frequently resists touching/being touched<br>rarely or never; almost always resists touching/being touched	*his slow, gentle hand and shoulder massage. RV01*<br>*3. She was very sleep but even though her eyes were closed she liked the contact, massage etc. She spoke and sang with eyes closed:) FR02*<br>*4. Last session she was very tired but enjoyed her massage, She likes to look at the magazine, also [to do] knitting wool. FR05*<br>*5. FR06 was enjoyed, face, hands and feet massage.*<br>*6. The resident refused to have face massage but was comfortable with hands and feet massage. FR07*<br>*7. Loves the one on one attention. Responds positively. Loves the Namaste attention. We read a lovely book together, enjoyed the massage. Loves facial massage. FR05*<br>*8. Loved a hand and face massage and enjoyed the Christmas movie. FR05*<br>*9. Loves facial massage FR05*<br>*10. Was very tired today but*	interaction with others<br>____ 3. half the time; never initiates interaction, but does not resist interactions with others<br>____ 4. less than half the time; often or frequently resists interaction with others<br>rarely or never; almost always resists interaction with others	*was awake later. As I started massaging him. He enjoyed the bubbles and music. Only eye movements.*<br>*4. Sometimes open eyes, looking around RV01*<br>*5. Resident was awake throughout the session & enjoyed it. RV01*<br>*6. Awake beginning of the program then slept but he enjoyed bubbles. Looking around. Eyes movement. RV01*<br>*7. Mostly she chat together and enjoys the bubbles, Happy. RV02*<br>*8. Communication and colouring, listen music enjoyed bubbles. Little cat, happy, calm*<br>*9. Communication, listening music.?? RV02*<br>*10. She was observant, enjoyed bubbles, engaged with bubbles, enjoyed brushing the little cat, chatting with staff, reading magazines, enjoyed listen to music. RV02*<br>*11. Was awake over the session and was enjoying the music and bubbles. She was calm looking at the magazine. She was reacting to my conversation and was happy. RV02*<br>*12. Back to bed, bedside massage. Happy, communication, enjoyed*

	enjoyed her massage. FR05<br>11. Enjoys coming to Namaste Care; really enjoys the massage. FR06[resident died during intervention]		bubbles. RV02<br>13. Awake over the session, enjoyed bubbles, music. RV02<br>14. She singing, communication, happy, calm. RV02<br>15. Happy, watching TV RV02<br>16. Happy, calm communication RV02<br>17. Happy, calm, watching movie. RV02<br>18. Communication, in the beginning of the session. Happy, watching movie, RV02<br>19. Engaged throughout the session RV04<br>20. Engaged RV04<br>21. Engaged throughout the session RV04<br>22. At the last session, while she was provided bedside care, she was alert and communication with staff. RV04<br>23. Bedside care provided, at the beginning of first NC, she was awake and then calm, sleeping over the two next session, to awake at the last session RV04<br>24. She is enjoying & relaxing during the care but after hour she also like to go action in café. RV04<br>25. Talked a lot and laughed a lot. Coloured in.<br>She is so happy the care. FR01<br>26. Some classical music enjoys

			the interaction and pampering but asks for nothing. FR01<br>27. *Very happy enjoys all of the activities & conversations. FR01*<br>28. *She was happy and talked about her journey to London FR02*<br>29. *Very happy. Had wonderful conversations all about her past. What triggered her past was I read poetry to her. She used to read poetry. FR02*<br>30. *I feel she enjoys other people company. FR02*<br>31. *Looks so happy today doing the care. She allowed everything. She did some colouring.FR02*<br>32. *She is happy & relaxed doing all care. She is so chatty and looks so happy. FR02*<br>33. *Was very talkative today enjoyed the music and movie. FR02*<br>34. *Enjoyed her hand massage & hair brushed. Christmas music playing, she enjoyed the music. FR03*<br>35. *Talking about her past. Likes to look at magazine. FR03*<br>36. *Enjoyed a massage and we read a lovely little book called And Wisdom comes quietly. We also sang Christmas carols. FR04*<br>37. *Only wanted her feet massaged today. We had Christmas music playing, tried*

			a bit of colouring in. Read all about London. FR04<br>38. *Today we had a quiet time with an Audrey Hepburn movie, enjoyed it a lot. FR04*<br>39. *Enjoys today's session. Was a little tired but apart from that quite happy. FR05*<br>40. *Sleepy today so just a quick session. Enjoyed the music. FR05*<br>41. *Was a latecomer today but enjoyed meeting the other ladies. FR05*<br>42. *Enjoyed all care. She likes to look magazine also to do knitting wool FR05*<br>43. *She loves the Namaste it's the one opportunity she get to have one on one. She loves female company, and the talk. FR05*<br><br>*All residents were seen to interact and engage with activities, or staff or were perceived as enjoying the 'company' of others. Staff described interactions as; 'loves', 'enjoyed'.*
Appears emotionally calm and comfortable<br>____ 1. most of the day<br>____ 2. more than half the day<br>____ 3. half the day<br>4. less than half the day	1. *In the beginning, he was sleepy but after a while he was awake during the session but was sitting expressionless, ideally [sic] looking at the staff. RV1*		

rarely or never	2. *Happy, calm, eyes looking around, enjoyed bubbles. RV1*<br>3. *At beginning, he was sleeping but after some time awake. Looking around. Calm. RV01*<br>4. *During the session, mostly slept, but sometimes open eyes looking around. Happy, calm. RV01*<br>5. *Awake over the session and enjoyed bubbles. Look around moving eyes. Happy and calm. RV01*<br>6. *She happy, calm middle of session and started chatting and laughing. Enjoyed the music RV02*<br>7. *Happy and calm, enjoyed the music, bubbles, little cat. RV02*<br>8. *Reading magazine, bubbles, listen music, happy and calm. RV02*<br>9. *Communication, colouring reading magazine, bubbles, she brush cat, happy, calm. RV02*<br>10. *Happy, calm, relax, enjoyed music bubbles, brush little cat, communication during the session. She was happy RV02*<br>11. *Happy, calm communication. RV02*<br>12. *Sleeping at beginning of the session then wake up, watching the movie, happy,*		

	calm until the end of the session. RV02<br>*13. Happy, calm, communication. RV02*<br>*14. Relaxing and mostly slept. FR01*<br>*15. She is bit agitated but after having massage and drinks [is] looks happy and relaxed. FR02*<br>*16. Very happy, content. FR02*<br>*17. Very content and calm. Beautiful music very calm FR03*<br>*18. She enjoyed the care, she is happy & relaxed FR04 She is enjoying & relaxing during the care FR04*<br>*19. Had a nice relaxing time today in the sunshine. FR04*<br>*20. Looks happy &relaxed during the care. She likes to roll wool to make ball. She enjoys with ice pole & she is so relaxed. FR05*<br>*21. Enjoyed her experience today. Very relaxed FR05*<br>*22. She enjoys all care, massage, drink & ?; she loves to do knitting wool.FR05*<br><br>*Residents were perceived as calm and relaxed. Some were engaged in an activity eg. Rolling wool, or simply looking around whereas they had been*		

	previously sleepy.<br><br>*RV01, RV02, RV04, FR01, FR02, FR03, FR04, FRO5*		
_______ ***Total Score*** (sum of all items: scores range from 11 to 55 with lower scores representing higher quality of life)	*For some session, she was calm in the beginning and then started to communicate with others. RV02*	**Resident Name _____________**<br>**Date:**	
Knowledge/familiarity of caregiver(s) with the resident<br>_____ 0 Very familiar; provides daily care<br>_____ 1. Somewhat familiar, often provides some care<br>_______ 2. Not very familiar; only minimal contact		***Quality of Interview*** *(Interviewer's Professional Judgement)*<br>_____ 0 Interview appeared valid<br>_____ 2. Some questions about interview, but probably acceptable<br>_______ 3. Information from interview of doubtful validity	
FR06 notified Lavender scent			

Unclear code:

1. *She is ok with the NC for the first 30 minutes then was wondering around. FR07*
2. *A bit tired and sleepy in the middles of the session. Did not want to be touched her hair & face.*
3. *Slept, communication, not respond.*
4. *Responsive to session bedside care*
5. *Watched some Faulty Towers as she was being pampered.*
6. *This session residents watched some faulty towers while they were being pampered*
   This session wanted to watch DVD while be pampered. After here resident likes to go action in café.
